# The Treatment of Epilepsy by Belladonna

**Published:** 1863-08

**Authors:** J. S. Ramskill

**Affiliations:** Assistant-Physician to the London Hospital, and Physician to the Hospital for Epilepsy and Paralysis


### THE TREATMENT OF EPILEPSY BY BELLADONNA.

By Dr. J. S. RAMSKILL, Assistant-Physician to the London Hospital, and Physician to the Hospital for Epilepsy and Paralysis.

Concerning the treatment by, and action of, belladonna in epilepsy, I will give you, in a short compass, the results of my experience in its use. First, you must not always, nor even usually, look for immediate and palpable beneficial results. The number of fits at first may not lessen in equal times; very frequently, the reverse obtains; and you may expect, for three or four weeks after commencing it, even in the most appropriate cases, a complaint that the patient gets worse; but after six or eight weeks, if any amelioration occur, it will be decided and progressive. At first the dose should be very small, and gradually augmented until the pupil shows signs of its action, and the patient complains of both alteration in sight and dryness of throat. Having obtained this result, and maintained it for some weeks, the dose may be gradually diminished; but its effects on the eye and throat are not to be so diminished as to become imperceptable to the patient, but only so far lessened as to cease causing absolute discomfort. The other toxic effects of belladonna are wholly uncalled for. Patients vary greatly, both as to susceptibility in the action of the drug, and in other respects. The annoyance as to dry throat and disturbed vision, which, at the expiration of a month, may be said to be unendurable, will now and then cease, the dose being the same, or even slightly increased; but I may remark, these cases always improve most rapidly. I prefer to give the drug in an eighth of a grain dose three times, or only twice, daily, for a week; then a quarter of a grain for fourteen days; a third for the next fourteen days, at which time its physiological action will in most cases be manifest. I think it wise to halt at this dose for two months or three months, slightly increasing the dose if the patient shows diminished susceptibility to its influence, decreasing it if the reverse happens, and then gradually dropping it to the quantity first administered. I have given as much as four grains for a dose, but very rarely. I think it imperative to say, that I have never been able to give in epilipsy the large doses which Dr. Fuller has succeeded in administering in other diseases of a convulsive character. In this remark I am supported by the authority of my colleague, Dr. Brown-Séquard, who has arrived at the same conclusion. One objection to the use of belladonna, when you cannot see your patient at regular intervals, arises from its uncertainty of strength and corresponding difference of action. To those who wish to use a preparation of uniform strength, having similar, and, in some cases, improved properties of belladonna, the salts of atropia are now easily procurable. The best of these is the valerianate of atropia; the commencing dose a-hundred-and-twentieth of a grain. Hitherto, I have preferred belladonna, having had a strong desire to find what it could and, if possible, what it could not accomplish in the treatment of epilepsy. It is right to say, there are different methods of administering belladonna. Trousseau gives a centigramme of the extract and an equal quantity of the powder of belladonna for the first month, in the evening of each day. He gives it at this time because of the frequent nocturnal character of epilepsy, and partly because of the disagreeable effect on the sight and throat during its early administration. During the second month, he gives two such pills at the same time, and during the third month, three pills. If, at the end of six or nine months, the frequency of the fits is decreased, he increases the dose. He asserts that, of 120 patients, he has cured twenty. A most important question now arises,—Do we know anything of the nature of the action of belladonna beyond the empirical results obtained in treatment? If a drop of solution of belladonna or atropine be dropped on the foot of a frog properly prepared, and fixed on the field of a microscope, the bloodvessels will be seen to contract, and they will remain in this condition for a considerable time. For comparing the action of opium, a solution of the latter, similarly prepared, was applied to another part, and the vessels were immediately dilated. Now, belladonna, internally administered in medicinal doses, causes, first, dilitation of the pupil, with dimness of vision; secondly, dryness of throat and difficulty of swallowing; thirdly, increased tone of involuntary muscle; fourthly, it relaxes the bowels, and cures incontinence of urine, arising from weak sphincter vesicæ. As dilatation of pupil is one of the earliest phenomena, let us see if we can account for it. There are two sets of fibres in the iris. It is well known that the sympathetic is the motor nerve of the external longitudinal fibres of the iris, which radiate from the centre to the circumference. The branch of nerves supplying these fibres comes from the cervical ganglia of the sympathetic. Excitation of this nerve, from any cause, will cause a contraction of these longitudinal fibres, and a corresponding dilitation of pupil. There is also a circular set of fibres immediately surrounding the margin of the pupil. This set is under cerebral control; that is to say, its motor supply comes from a branch of the third nerve. Any irritation in the brain or along the trunk of the nerve, or an excitation by light on the retina acting in a reflex manner, will stimulate this branch of the third to action, and cause contraction of pupil.

But we may have dilitation of pupil without increased action of the sympathetic; it may be acting normally, then the third nerve must be supposed dificient in power. This is a common result observed in compression of brain. On the other hand, contraction of pupil may be present without abnormal activity of the third being necessarily supposed. This condition is invariably produced by section of the sympathetic in the neck. Dilitation of pupil may, in short, depend upon the action of the sympathetic being in excess, or in diminished power of the cerebral nerve. In epilepsy it is easy to observe, from collateral symptoms and the general condition of the patient, that dilated pupil, when it exists, which is much rarer than a normal condition, is usually caused by an active sympathetic overpowering the third nerve. The same dilitation may be observed in most convalescents after acute disease, and in most affections involving extreme debility; but here it would be more correct to say, that the dilitation was rather the effect of a compressed condition of the third cerebral nerve accompanying a normal sympathetic, than of an active sympathetic accompanying a normal condition of the cerebral nerve. I have said the branches of the sympathetic nerve which go to the iris, come from the cervical sympathetic. Dr. A. Walker, with Professor Budge, have made experiments, which seem to prove that the nerve fibres of the cervical sympathetic, which go to the iris, originate from the spinal cord between the sixth cervical and the fourth dorsal vertebræ. Dr. Brown-Séquard has ascertained that the origins of the fibres of the sympathetic going to the iris are still more extended. I have mentioned that division of the cervical sympathetic allows the uncontrolled third cerebral nerve to contract the iris. Dr. Brown-Séquard has shown that a section of the spinal cord, as high as the level of the fifth cervical, or as low as the ninth or tenth dorsal vertebræ, affects the iris in the same manner, but in a less degree than section of the sympathetic. On the other hand, Schiff has shown that some of the fibres animating the iris ascend the cervical part of the spinal cord, and most probably go up to the medulla. I may also say here, that the sympathetic is the motor nerve of the bloodvessels, supplying various parts of the head. It is especially interesting to know the origin of these vaso-motor nerves, especially in relation to loss of consciousness, the initial movement of a fit of epilepsy, and also in regard to the pathology of the *petit-mal*, as well as the great light such knowledge would throw on the action of belladonna in epilepsy. Dr. Brown-Séquard discovered some years ago that the motor nerves of the bloodvessels going to various parts of the head, come out chiefly from the spinal cord by the roots of the last cervical and first and second dorsal nerves. He thinks, however, their real place of origin to be partly the spinal cord, partly the higher portions of the encephalon, but chiefly the medulla oblongata and the neighboring parts of the encephalon. In the case of R. P., it will be remembered the ferrum candens was applied to each side of the spine, opposite the last cervical and first dorsal vertebræ. The reason will now be apparent. The vaso-motor nerve fibres are able to contract the bloodvessels directly, when excited. We hope, by frequently cauterizing the tissues opposite the seat of exit of these nerves from the spine, to effect some change in the nutrition of the parts to which these nerves are distributed. We can now understand the nature of the action of belladonna in producing dilation of the pupil; and from its effect on the iris, we can deduce a strong probability of the nature of its action in epilepsy. It is a stimulant to the sympathetic, the motor-nerve of the bloodvessels, and it is only on this supposition we can account for the other physiological effects of the drug.

I would add, although experience shows belladonna is one of the most powerful contractors of the bloodvessels of the spinal cord and its membranes, it has a comparatively feeble action on those of the brain. I speak of its administration in medicine, —not in poisonous or fatal doses. Hence arises its extraordinary adaptability in epilepsy, where we have dilatations of vessels or turgescence in the medulla and its neighborhood; of its still more marked efficacy in inflammation, and congestion of the spinal cord and its membranes; as well as of its comparative inutility (administered alone,) in those cases of morbid activity of brain, connected, as we think, with more or less congestion of grey matter, in some forms of incipient insanity, associated with sleeplessness and suicidal tendency, as well as in some other cerebral diseases.—*Medical Times and Gazette.*

